# Menthol and initiation of cigarette smoking

**DOI:** 10.1186/1617-9625-9-S1-S4

**Published:** 2011-05-23

**Authors:** Joshua Rising, Kristina Wasson-Blader

**Affiliations:** 1 Center for Tobacco Products, U.S. Food and Drug Administration, Rockville, MD, USA; 2 KWB Health Communications, Inc, USA

## Abstract

The use of tobacco products would not continue without the initiation of their use by youth and adults. Since the vast majority of cigarette smokers begin smoking by age 25, understanding the role of menthol cigarettes in the initiation of smoking in youth (under the age of 18) and young adults (aged 18–25) is especially relevant. Data demonstrate that menthol cigarettes are disproportionately used by youth and young adults. This review seeks to examine what role, if any, menthol plays in the initiation of cigarette smoking. Overall, there is a paucity of data on this topic. The data that do exist suggests that youth who have smoked for less than 1 year are more likely to smoke menthol cigarettes than youth who have smoked for more than 1 year. A lack of data prevents further conclusions on the role of menthol cigarettes in the initiation of smoking.

## Review

### Introduction

Initiation of cigarette smoking in the United States has continued at a high rate. According to a 2008 report from the Substance Abuse and Mental Health Administration, around 4,000 youth try their first cigarette each day [1] and approximately 1,000 youth become daily smokers. [2] Additionally, 2,800 young adults try their first cigarette each day. [1] Other data have also demonstrated high rates of smoking initiation among youth and young adults. A report from the Surgeon General in 1994 [3] reviewed data from the National Household Surveys on Drug Abuse, which surveyed persons aged 30–39 about their recollections of the age of first cigarette and first regular smoking, and found that 62.2% of persons who had ever smoked daily tried their first cigarette before the age of 16. In addition, more than half of those who ever smoked daily began smoking daily before the age of 18 [3]. A study that used aggregated data from the 1990s examined the age at smoking initiation as recalled by individuals aged 26–50 years; this study revealed significant racial/ethnic disparities [4]. The authors found that the majority of White and Latino smokers began smoking by the age of 18, while the majority of Black/African American and Asian/Pacific Islander smokers began smoking between the ages of 18 and 25. While these reports do not distinguish between menthol and non-menthol cigarette smokers, they do provide data that demonstrate that the majority of smokers begin smoking as youth and young adults.

Despite acknowledgement of the importance of understanding why individuals initiate cigarette smoking, there is limited literature on the impact of menthol on the initiation of cigarette smoking. This article reviews the available literature on menthol and the initiation of cigarette smoking and explores the following questions:

• Do menthol cigarette smokers start smoking earlier in life than non-menthol cigarette smokers?

○ Does this vary by race/ethnicity?

• Is there a higher use of menthol cigarettes among recent initiations (e.g., youth who have been smoking for less than 1 year)?

○ Does this vary by age or race/ethnicity?

• Does the smoking of menthol cigarettes alter progression from first cigarette(s) toward becoming a regular smoker?

• Did current smokers initiate smoking with menthol cigarettes and then switch to non-menthol cigarettes?

• What information do published reports on publicly available internal tobacco industry documents contain on this topic?

Summarized in this review are 10 articles found to have either direct relevance to these questions, or were used to provide relevant background information. Many of these articles were identified through a review of the literature conducted by the National Cancer Institute in 2009, published as “Bibliography of literature on menthol and tobacco” (http://cancercontrol.cancer.gov/tcrb/documents/menthol_bibliography_508.pdf). Search terms used were menthol cigarette(s); mentholated cigarette(s); menthol tobacco; mentholated tobacco; menthol smoker(s); menthol AND the following terms: addiction, nicotine, marketing, cancer, biomarkers, asthma, cardiovascular disease, heart disease, vascular disease, chronic obstructive lung disease, respiratory, environmental tobacco smoke, national health, health disparities, and minority health. Additional searches and sources, such as those identified through review articles, identified additional articles that were included as appropriate.

Of those articles that are in the NCI Bibliography but were not included, most were not directly relevant to this topic (e.g., they studied menthol as a chemical independent from tobacco smoke exposure, did not evaluate menthol as a separate variable). Some of those articles, however, were used to provide background information. Animal or in vitro research was included only to help explain human findings. Although a few review articles were used to make general statements and/or provide background information, most were not included in deference to original sources. Published abstracts were not included out of concern that, due to the lack of details, those studies could not adequately be assessed.

### New youth and young adult smokers

A study by Hersey et al [5] examined data from the 2002 National Youth Tobacco Survey regarding the duration of smoking and menthol cigarette use. Middle school students (grades 6–8) who had been smoking less than 1 year were significantly more likely to smoke menthol cigarettes than were middle school students who had been smoking more than 1 year (62.4% vs. 53.3%, p < .002). A similar, though not statistically significant, pattern was found for high school students (grades 9–12); 46% of the high school students who had been smoking for less than 1 year smoked menthol cigarettes, compared with 42% of students who had been smoking more than 1 year.

In National Survey on Drug Use and Health aggregated data from 2004 to 2008, among smokers who had been smoking less than 1 year, the proportion who smoked menthol cigarettes was greater than the proportion who smoked non-menthol cigarettes for both youth (49.2% vs. 43.8%) and young adult smokers (40.2% vs. 36.4%), providing further indications of greater use of menthol cigarettes among recent initiators; however, statistical significance was not reported [6].

**Figure 1:**
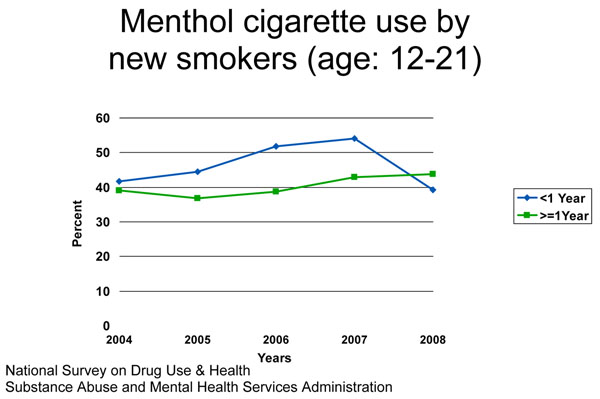
Menthol cigarette use by new smokers (age: 12-21)

Unpublished data from the years 2004 to 2008 on the use of menthol cigarettes by young smokers (aged 12–21 years) from the National Survey on Drug Use and Health [6] show a similar pattern (see Figure 1), with menthol cigarettes being used more among new smokers (smokers for less than 1 year) than among experienced smokers (smokers for more than 1 year). Although this pattern reversed in 2008, more data points will be needed to assess whether this is an aberration or the beginning of a new pattern.

### Age of initiation

The age at which cigarette smoking (menthol or non-menthol) was initiated has been evaluated in two studies [7,8]. Hymowitz et al [7] reported on the baseline characteristics of the cohort enrolled in the National Cancer Institute–funded Community Intervention Trial for Smoking Cessation (COMMIT) trial. Enrollment for the COMMIT trial occurred in 1988. The researchers reported that there was no difference in the ages at which menthol cigarette smokers and non-menthol smokers started smoking. The Coronary Artery Risk Development in Young Adults (CARDIA) study also found both non-menthol smokers (n = 563) and menthol smokers (n = 972) smoked their first cigarette at an average age of 16 [8]. Both the Hymowitz et al and Pletcher et al studies used retrospective questions to assess “age started smoking” (Hymowitz et al) and “age smoked first cigarette” (Pletcher et al).

### Early menthol cigarette use and subsequent cigarette use and dependence

Only one study was identified that looked at whether early menthol cigarette use is predictive of nicotine dependence. A longitudinal study by DiFranza et al [9] followed seventh graders for 30 months and then analyzed data on the 237 individuals who had either inhaled a cigarette before the start of the study or who first inhaled a cigarette during the study period. Approximately half of these youth were able to report if their first cigarette had been a menthol one (among those who could remember, 42% reported that it was). There were no differences in reported reactions (nausea, dizziness, relaxation, irritation) to the first cigarette [9]. No studies examined whether the early use of menthol cigarettes affected an individual’s trajectory to regular smoking.

### Switching between menthol and non-menthol cigarettes

No studies were identified that directly addressed whether current smokers started smoking with menthol cigarettes and then switched to non-menthol cigarettes. A 1989 study, however, examined the switching behavior of 29,037 current smokers who attended the Kaiser Permanente Medical Care Program between 1979 and 1986. Study participants were followed for a mean of 4.5 years [10]. Of the 1,688 Black/African American smokers under the age of 40 for whom follow-up data were available, the percentage of those who switched from non-menthol cigarettes to menthol cigarettes (14.6%) was much higher than the percentage of menthol cigarette smokers who switched to non-menthol cigarettes (3.6%). Using a proportional hazards model adjusting for age and sex, the researchers found that non-menthol cigarette smokers were 4.2 times more likely to switch to menthol cigarettes than for menthol cigarette smokers to switch to non-menthol cigarettes.

One other study described switching behavior. Pletcher et al [8] found no difference in the percentage of young adult smokers who had switched types of products at the 15-year follow-up in the CARDIA study; 12% of those who originally preferred menthol cigarettes had switched to non-menthol cigarettes, and 11% of those who originally preferred non-menthol cigarettes had switched to menthol cigarettes.

### Research on publicly available internal tobacco industry documents on menthol and initiation

Two reviews of internal tobacco company documents on the topic of menthol cigarettes were performed by Kreslake et al [11,12]. The authors cite a tobacco company document that found a benefit to including menthol in cigarettes in order to make the smoking experience more palatable for inexperienced smokers. “First-time smoker reaction is generally negative…Initial negatives can be alleviated with a low level of menthol” [12]. They also found available documentation that tobacco companies were aware that newer smokers preferred menthol cigarettes with a lower level of menthol than established smokers. According to Kreslake et al, “the author of an internal Brown and Williamson memo observed that Kool’s menthol level may be considered too high for new smokers, and that a successful ‘starter’ cigarette would need to include a low tobacco taste, low impact and irritation, low tobacco aftertaste and low menthol content” [11].

## Conclusions

Limited data exist regarding the role of menthol in the initiation of smoking. Furthermore, there are significant limitations to the existing studies. Some of the studies that used retrospective data and adult recollections of smoking initiation may not provide an accurate representation of the product used. Only three studies followed individuals prospectively, and two of these studies began following individuals after smoking had been initiated. The third of these studies [9] had a small sample size.

However, Hersey et al [5] specifically addressed the issue of potential incomplete knowledge of menthol status of cigarettes used by younger smokers. These researchers examined the data based on several different definitions of “menthol cigarette smoker” and found that the results were similar regardless of the definition, indicating that the rate of misclassification of menthol status of cigarettes by new users was low [5].

Even with the above limitations, however, the existing literature does support the following conclusions:

• The vast majority of individuals who become regular smokers begin smoking as youth or young adults.

• Menthol cigarettes are widely used among youth who have smoked for less than one year and are used less frequently by youth who have smoked for more than one year.

• Although limited data are available, there appears to be no differences in age of initiation between those who start smoking with menthol cigarettes and those who start smoking with non-menthol cigarettes; however, because the data are retrospective, they reflect initiation rates 16-30 years ago, not current patterns.

• Results are inconsistent regarding the frequency and direction of switching between menthol and non-menthol cigarettes.

• No data exist on whether menthol cigarette use alters the trajectory from initiating cigarette use to regular smoking.

• Reviews of publicly available internal tobacco industry documents suggest an industry awareness of the appeal of menthol cigarettes to newer smokers.

## Competing interests

The authors declare that they have no competing interests.
